# GABA_A_ receptor function is enhanced by Interleukin-10 in human epileptogenic gangliogliomas and its effect is counteracted by Interleukin-1β

**DOI:** 10.1038/s41598-022-22806-9

**Published:** 2022-10-26

**Authors:** Gabriele Ruffolo, Veronica Alfano, Alessia Romagnolo, Till Zimmer, James D. Mills, Pierangelo Cifelli, Alessandro Gaeta, Alessandra Morano, Jasper Anink, Angelika Mühlebner, Annamaria Vezzani, Eleonora Aronica, Eleonora Palma

**Affiliations:** 1 Department of Physiology and Pharmacology, Istituto Pasteur-Fondazione Cenci Bolognetti, University of Rome Sapienza, Rome, Italy; 2 IRCCS San Raffaele Roma, Rome, Italy; 3 Department of (Neuro)Pathology, Amsterdam UMC Location University of Amsterdam, Amsterdam Neuroscience, Meibergdreef 9, Amsterdam, The Netherlands; 4 Department of Clinical and Experimental Epilepsy, UCL Queen Square Institute of Neurology, London, UK; 5 Chalfont Centre for Epilepsy, Chalfont St Peter, UK; 6 Department of Applied Clinical and Biotechnological Sciences, University of L’Aquila, L’Aquila, Italy; 7 Department of Human Neuroscience, University of Rome Sapienza, Rome, Italy; 8 Department of Pathology, University Medical Center Utrecht, Utrecht, The Netherlands; 9 Department of Neuroscience, Istituto di Ricerche Farmacologiche Mario Negri IRCCS, Milan, Italy; 10 Stichting Epilepsie Instellingen Nederland, Heemstede, The Netherlands

**Keywords:** Neuroscience, Physiology

## Abstract

Gangliogliomas (GGs) are low-grade brain tumours that cause intractable focal epilepsy in children and adults. In GG, as in epileptogenic focal malformations (*i.e.,* tuberous sclerosis complex, TSC), there is evidence of sustained neuroinflammation with involvement of the pro-inflammatory cytokine IL-1β. On the other hand, anti-inflammatory mediators are less studied but bear relevance for understanding seizure mechanisms. Therefore, we investigated the effect of the key anti-inflammatory cytokine IL-10 on GABAergic neurotransmission in GG. We assessed the IL-10 dependent signaling by transcriptomic analysis, immunohistochemistry and performed voltage-clamp recordings on *Xenopus* oocytes microtransplanted with cell membranes from brain specimens, to overcome the limited availability of acute GG slices. We report that IL-10-related mRNAs were up-regulated in GG and slightly in TSC. Moreover, we found IL-10 receptors are expressed by neurons and astroglia. Furthermore, GABA currents were potentiated significantly by IL-10 in GG. This effect was time and dose-dependent and inhibited by blockade of IL-10 signaling. Notably, in the same tissue, IL-1β reduced GABA current amplitude and prevented the IL-10 effect. These results suggest that in epileptogenic tissue, pro-inflammatory mechanisms of hyperexcitability prevail over key anti-inflammatory pathways enhancing GABAergic inhibition. Hence, boosting the effects of specific anti-inflammatory molecules could resolve inflammation and reduce intractable seizures.

## Introduction

Gangliogliomas (GGs) are the most frequent tumor type among developmental low-grade brain tumors which are well-recognized causes of intractable focal epilepsy in children and young adults^1,2^. Accordingly, epileptic seizures are reported in 80–100% of patients with GG compared to 30% in malignant gliomas^3^. However, the pathophysiological mechanisms of GG epileptogenicity are still poorly understood^4,5^.

Due to their strong association with epileptic seizures, it was suggested that GGs are endowed of intrinsically altered synaptic functions. This clinical feature aligns with recent findings that the oncogenic BRAF somatic mutation in GG elicits hyperexcitability^6^ that is mediated by RE1-silencing transcription factor, a master regulator of ion channels and neurotransmitter receptors in epilepsy^3^, and by the activation of the epileptogenic Akt/mTOR signaling^7,8^.

One factor likely contributing to GG epileptogenicity relates to neuroinflammation that is described in these lesions^4,9^, since this phenomenon is involved in both epileptogenesis and ictogenesis^10^.

Indeed, the expression and receptor signaling of various cytokines undergo changes in epileptic foci, and cytokine levels are often modified in serum and cerebrospinal fluid of patients with epilepsy^11^. Some of these molecules play a role in seizure generation in animal models by modifying the activity of voltage-gated or receptor-coupled ion channels^12,13^, and by inducing transcriptional changes of genes involved in synaptic transmission and epileptogenesis^10,13^. In particular, the prototypical inflammatory cytokine interleukin-1β (IL-1β) plays a pivotal role in ictogenesis and epileptogenesis both in experimental models of epilepsy^14–16^ and patients.

Our investigation stemmed from the observation that the developmental brain tumours, such as GG and TSC cortical tubers represent common causes of drug-resistant focal epilepsy with early seizure onset^5^. In addition, recent advances highlight the involvement of different, but also converging, epileptogenic mechanisms including the activation of mTOR pathway as well as a sustained inflammatory response in both these lesions with the involvement of the pro-inflammatory cytokine IL-1β^17^.

The anti-inflammatory cytokine interleukin-10 (IL-10)^18,19^ has attracted attention in epilepsy as master regulator of glial cell inflammatory phenotypes^20^. Moreover, IL-10 was shown to reduce IL-1β production and inflammasome activation in experimental epilepsy^21^ and attenuated behavioral changes induced by chronic administration of IL-1β in rats^22^. However, scarce information is available on the effects of IL-10 on synaptic transmission and whether IL-10 modulates the neuronal activity as reported for IL-1β^23^ Cytokines and chemokines may affect Ca^2+^ permeability of NMDA and AMPA receptors^13^ and regulate GABA_A_ receptors (GABA_A_Rs) trafficking^24^. Interestingly, while IL-1β decreased the amplitude of GABA-evoked currents^23^, the chemokine fractalkine (CX3CL1) reduced the GABA current desensitization in temporal lobe epilepsy (TLE), thus resulting in opposite functional effects on GABA neurotransmission^25^. This evidence suggests that the net effect of neuroinflammation on neuronal network excitability likely depends on the balance between the action of individual cytokines/chemokines and how their effects are compensated for by anti-inflammatory mechanisms^13,26^. Here, we studied the expression of IL-10 and IL-1β related genes and proteins by transcriptomic analysis and immunohistochemistry in GG as compared with TSC-cortical tubers, highly epileptogenic focal malformations. We performed electrophysiology experiments to study IL-10 and IL-1β effects on GABAergic neurotransmission in order to shed light on the effects of anti-inflammatory and pro-inflammatory stimuli on neurotransmission in epileptogenic lesions.

## Results

### Differential expression analysis of* IL-1β* and* IL-10* pathway related genes

**Figure 1:**
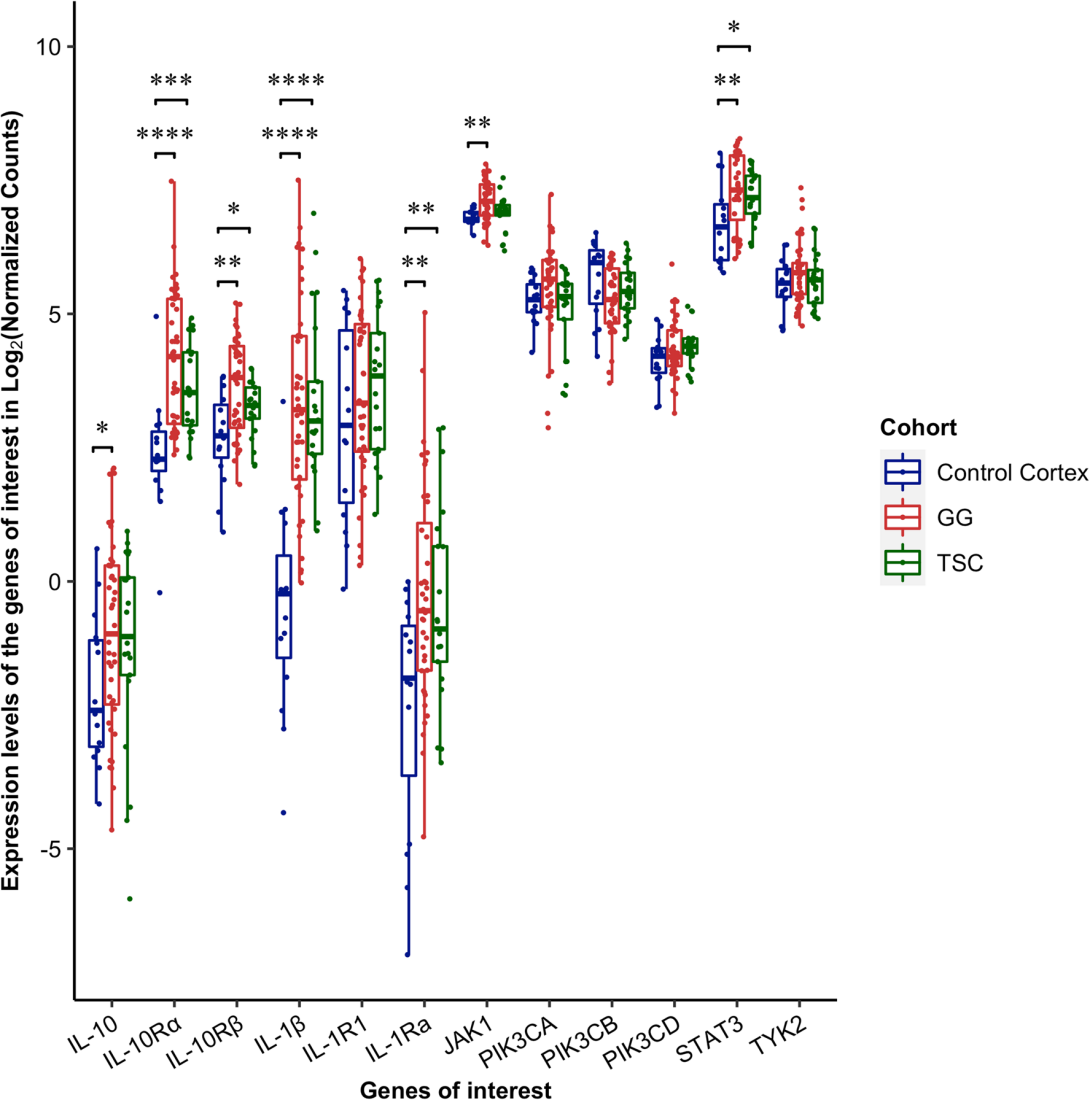
Expression of genes of interest in controls, GG and TSC. RNAseq data indicate significant up-regulation (*adjusted p value* = *0.05*) of *IL-10Rα*, *IL-10Rβ*, *IL-1Ra*, *IL-1β* and *STAT3* in both GG and TSC. In addition, there is a significant upregulation of *IL-10* and *JAK1* in GG. *IL-10* downstream signaling protein such as *TIK1*, phosphokinases (*PIK3CA, PIK3CB, PIK3CD*) and *IL1-R1* did not show significant changes in either GG or TSC vs controls. * *p* < 0.05; ** *p* < 0.01; *** *p* < 0.001; **** *p* < 0.0001. A linear model was fit for each gene and moderated t-statistic was calculated after applying an empirical Bayes smoothing to the standard errors. Those genes with a Benjamini–Hochberg adjusted *p* value < 0.05 were considered significant. Differential expression analysis compared 21 TSC patients and 15 matched control cortices; 37 GG patients and 15 matched controls cortices.

The IL-10R complex includes the IL-10 binding subunit IL-10Rα and the accessory subunit IL-10Rβ responsible for recruitment of downstream signaling proteins^27^. IL-10 binding to its receptor leads to the activation of the proximal kinases JAK1-JAK2-TYK2 and subsequently of phosphokinases and the STAT3 system^18,27^. Therefore, we first carried out a differential gene expression analysis of mRNAs encoding several proteins involved in the IL-10 downstream signaling pathway (Fig. 1) in GG and TSC patients who underwent surgery for drug-resistant epilepsy and compared with control cortex cases (Supplementary Information).

We found that *IL-10* transcript was significantly upregulated only in GG patients (log_2_ fold-change, FC = 1.019) (Fig. 1) whilst its receptors (*IL-10Rα* and *IL-10Rβ*) and *STAT3* were upregulated in both GG (*IL-10Rα* log_2_FC = 1.775; *IL-10Rβ* log_2_FC = 0.956; *STAT3* log_2_FC = 0.622) and TSC (*IL-10Rα* log_2_FC = 1.212; *IL-10Rβ* log_2_FC = 0.534; *STAT3* log_2_FC = 0.527) (Fig. 1). As for *IL-10* transcript only *JAK1* showed significant overexpression in GG (log_2_FC = 0.303) (Fig. 1) whilst *TYK2* and the phosphokinases *PIK3CA, PIK3CB, PIK3CD* were not differentially expressed in either TSC or GG (Fig. 1).

The *IL-1β* and *IL-1Ra* (IL-1 receptor antagonist) were significantly upregulated in both TSC (*IL-1β* log_2_FC = 3.846; *IL-1Ra* log_2_FC of 1.872) and GG (*IL-1β* log_2_FC = 3.832; *IL-1Ra* log_2_FC = 2.097) (Fig. 1). Noteworthy, the ratio between *IL-1β*/*IL-1Ra* was shifted towards the pro-epileptogenic *IL-1β* (2.05- and 1.83-fold in TSC and GG, respectively) suggesting that the IL-1β signaling was not efficiently controlled by the required ~ 100-fold excess of IL-1Ra^28^. *IL-1R1* transcript showed no differential expression in epileptogenic lesions *versus* control tissue (Fig. 1).

### Cellular expression of IL-10Rα in GG and TSC

**Figure 2:**
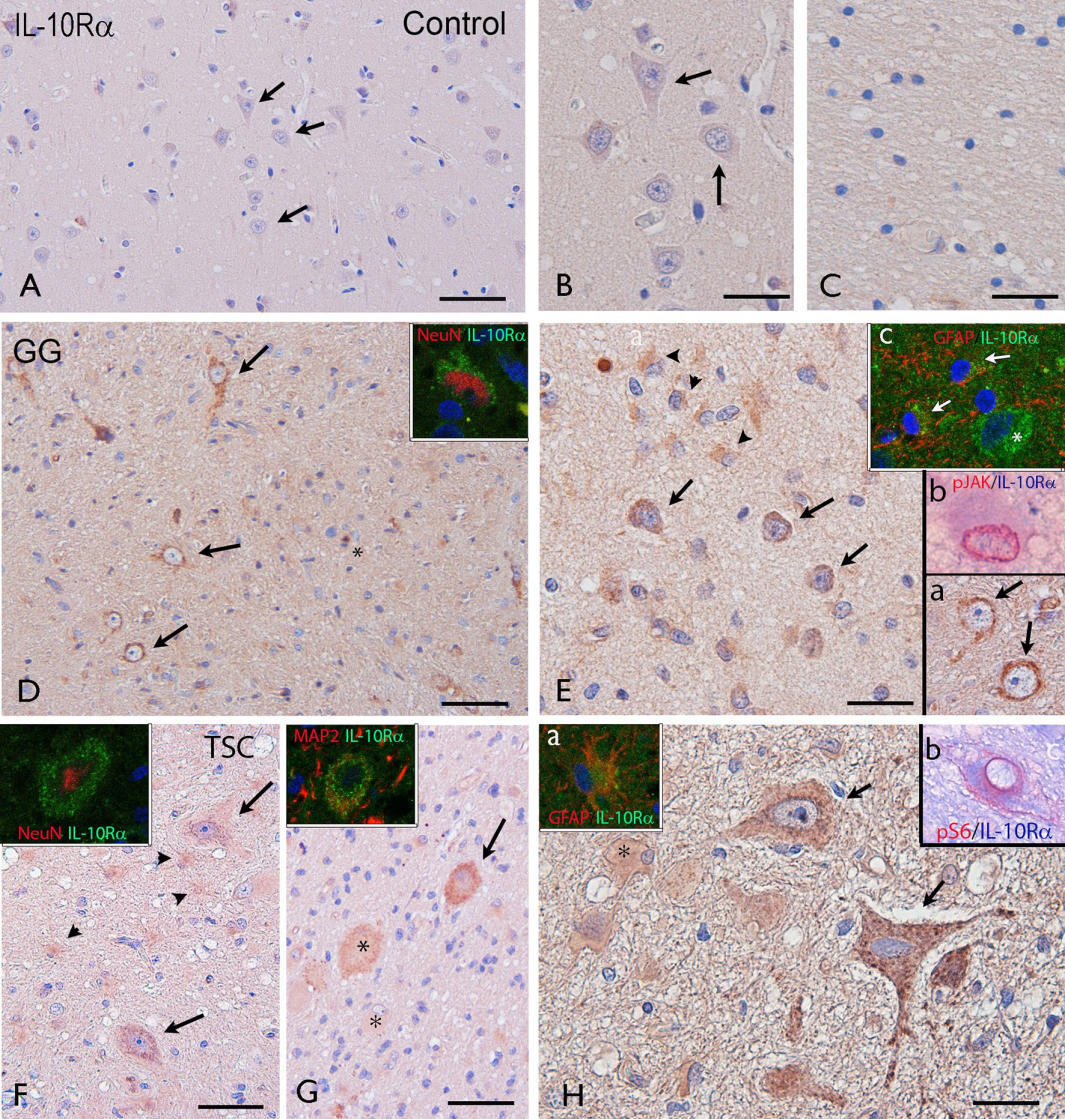
Cellular expression pattern of IL-10 receptor in GG and TSC. Representative photomicrographs of immunocytochemical staining for IL-10Rα. Sections are counterstained with haematoxylin. Panels (**A**–**C**): IL-10Rα immunoreactivity (IR) in control brain. No detectable neuronal or glial labelling is observed in normal cortex (**A**–**B**; arrows indicate neurons) and white matter (**C**). Panels (**D**–**E**): IL-10Rα in gangliogliomas (GG). IL-10Rα IR was observed in dysplastic neurons (arrows, D and insert-a in E); insert in D shows expression of IL-10Rα (green) in a NeuN positive (red) dysplastic neuron; insert-b in E shows co-localization of IL-10Rα (blue) with pJAK (red). IL-10Rα IR was observed in scattered astroglial cells (arrowheads in E); insert-c in E shows co-localization of IL-10Rα (green) with GFAP (red). Panels (**F**–**H**): IL-10Rα in tuberous sclerosis complex (TSC). IL-10Rα IR was observed in dysmorphic neurons (arrows and inserts in F and H); IR was also observed in astrocytes (arrowheads; insert in H-a) and few giant cells (asterisks) within the dysplastic area. Insert in (**F**) shows expression of IL-10Rα (green) in a NeuN positive (red) dysmorphic neuron. Insert in (**G**) shows co-localization of IL-10Rα (green) with MAP2 (red). Insert (a) in (**H**) shows co-localization of IL-10Rα (green) with GFAP (red). Insert (b) in (**H**) shows co-localization of IL-10Rα (blue) with pS6 (red) in a dysmorphic neuron. Scale bar: (**A**, **D**): 100 µm; (**F**, **G**): 80 µm; (**B**, **C**, **E**): 40 µm; (**H**): 30 µm. Details on the cohort used are reported in Supplementary Information.

In human control cortex, throughout all cortical layers and white matter, IL-10Rα immunoreactivity was not detectable in neurons or glial cells (Fig. 2A–C). In GG, IL-10Rα immunoreactivity was observed in dysplastic neurons and tumor astrocytes (Fig. 2D, E). Double-labelling showed IL-10Rα expression in neuronal cells (NeuN-positive) and in GFAP-positive astrocytes. In GG, IL-10Rα was also detected in pJAK-positive cells. In TSC, IL-10Rα immunoreactivity was observed in dysmorphic neurons as well as in astrocytes and in scattered giant cells (Fig. 2F–H). Double-labelling showed IL-10Rα expression in neuronal cells (NeuN- and MAP2-positive) as well as in GFAP-positive astrocytes and in dysmorphic neurons positive for pS6, a marker of mTOR activation. Semiquantitative analysis of IL-10Ra immunoreactivity is shown in Supplementary information.

### IL-10 effect on GABA_A_ mediated currents

We determined whether the up-regulation of IL-10 and related signaling was associated with an effect of IL-10 on GABAergic transmission.

First, we used oocytes microinjected with human cDNAs encoding for α1β2γ2 GABA_A_Rs (the most common receptor isoform in CNS^29^) or α4β2γ2 (expressing α4, one of the most relevant subunit mediating tonic inhibition^29^) to test whether IL-10 affects GABA currents (I_GABA_) by direct interaction with GABA_A_R.

**Figure 3:**
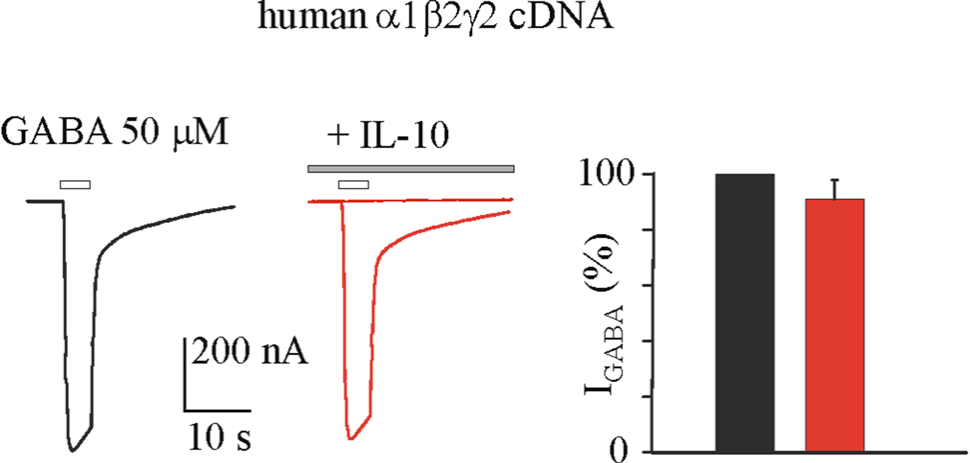
IL-10 effect on GABA current amplitude in oocytes injected with human α1β2γ2 cDNA. The bar-graph represents the mean and ± s.e.m. of the I_GABA_ amplitudes evoked from oocytes intranuclearly injected with α1β2γ2 cDNAs before (black) and after (red) the incubation with IL-10 (200 ng/mL, 3 h; n = 8; *p* > 0.05 by paired t-test).The I_GABA_ amplitudes recorded after the IL-10 incubation were normalized to the response obtained before exposure to IL-10 for each cell (range of current amplitudes: from 247.5 to 1119.0 nA), then averaged and expressed as a percent variation. Traces depict representative currents measured after 4 s application of GABA (white bar, 50 μM) in oocytes injected with α1β2γ2 cDNAs before (black trace) and after (red trace) IL-10 incubation (for 3 h). Grey bar on the right trace represents the block by 100 μM bicuculline (representative of 4 experiments).

IL-10 (100–200 ng/mL) co-applied with GABA 50 μM did not affect I_GABA_ (*not shown*). Similarly, IL-10 was ineffective on I_GABA_ when oocytes expressing α1β2γ2 were preincubated with the cytokine (100–200 ng/ml) for 3 h (50 μM GABA applied for 4 s; I_GABA_, 688 ± 431 nA before IL-10; 626.7 ± 379 nA, after 200 ng/ml IL-10; n = 8, Fig. 3). As previously shown^23^, these evoked currents were completely blocked by bicuculline (100 μM, Fig. 3). Similar results were obtained with oocytes expressing the α4β2γ2 after incubation with IL-10 for 3 h (50 μM GABA applied for 4 s; I_GABA_ 512 ± 270 nA before IL-10; 474 ± 203 nA, after 200 ng/ml IL-10; n = 6). Altogether these results show that there is not a direct interaction with α1 nor with α4 containing GABA_A_ receptors or an activation of endogenous signaling pathways.

**Figure 4:**
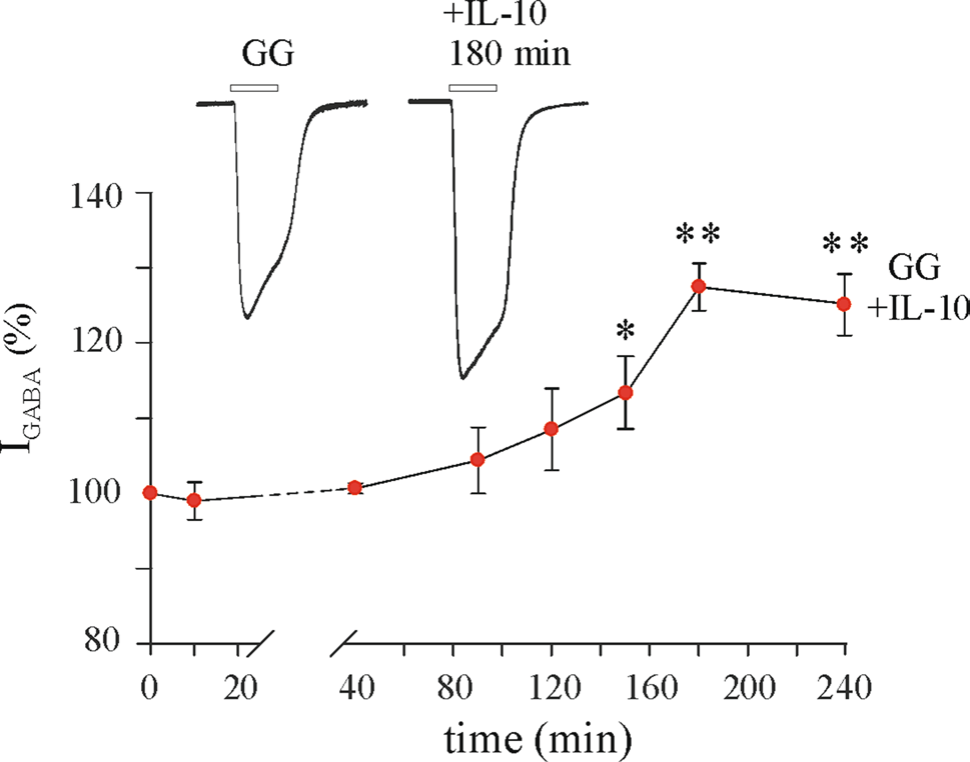
Time-course effect of IL-10 on GABA current amplitude in oocytes microinjected with GG tissue. GABA alone was applied at 250 μM at the beginning of each experiment (time zero) and at various time points after treatment with 100 ng/ml IL-10. Data (mean ± s.e.m.; n = 6–12 oocytes/time point; patients #8–12 in Table 1) represent the percentage increase of the peak amplitude induced by IL-10. Data were normalized to the mean current amplitude recorded at time zero (23.7 ± 8.6 nA, n = 12). *Inset*: Traces depict representative GABA currents at the indicated times. * = *p* < 0.05 by Wilcoxon signed rank test; ** = *p* < 0.01 by paired t-test.

**Figure 5:**
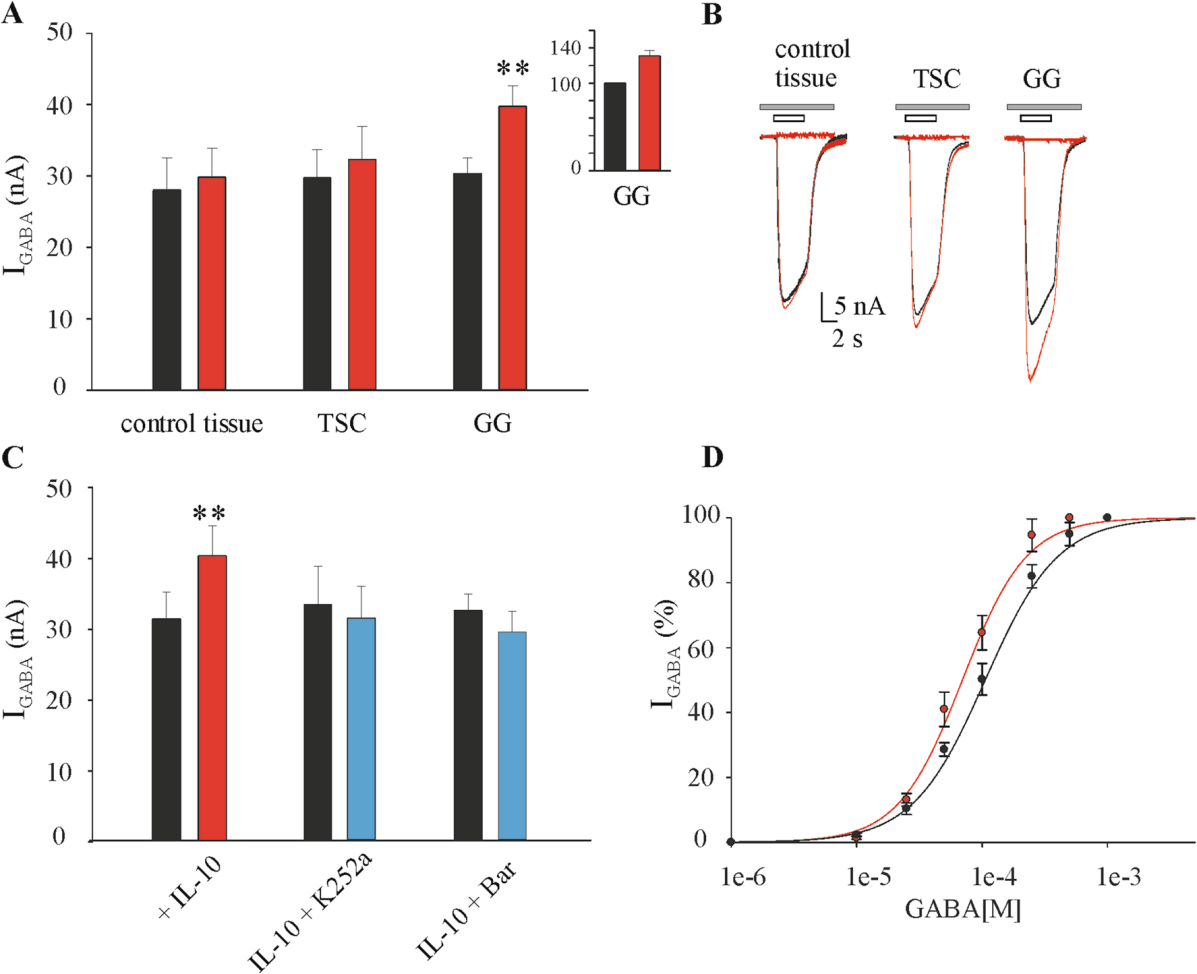
Characterization and blockade of IL-10 effect on GABA current amplitude. (**A**) Bar-graph of IL-10 effect on I_GABA_ current amplitude in oocytes microtransplanted with control tissue (n = 17), TSC (n = 24) and GG (n = 80) tissues. Data are expressed as mean ± s.e.m. *Inset*: I_GABA_ amplitude is expressed as percent increase above baseline (before IL-10 incubation, ranges of current amplitudes: control tissue, from 3.8 to 53.7 nA; TSC, from 7.7 to 73.7 nA; GG, from 5.5 to 84.0 nA). ** = *p* < 0.01 by paired t-test. (**B**) Representative superimposed current traces (GABA 250 μM, white bars) of control-, TSC- and GG-injected oocytes before (*black trace*) and after (*red trace*) incubation with IL-10 (100 ng/mL for 3 h). Grey bars represent the block by 100 μM bicuculline (representative of 3 experiments for each tissue) (**C**) Bar-graph shows the effect of incubation of K252a (2 μM, a broad-spectrum protein kinases inhibitor) or baricitinib (Bar 0.5 μM, a selective JAK1 and JAK2 inhibitor) with IL-10 (100 ng/ml). Black bar-graph represents the mean current value (nA) before incubation with IL-10 alone (red, n = 18) or in combination with the two blockers (blue, n = 8 for each blocker). ** = *p* < 0.01 by paired t-test (**D**) Dose–response curves of GABA (1 μM–1 mM) before (*black curve*) and after (*red curve*) incubation with IL-10 (100 ng/ml for 3 h) in oocytes microinjected with GG tissues (Patients # 8–10, Table 1). Averaged EC_50_ were 107.0 ± 9.7 μM, n_H_ = 1.4 ± 0.10, before IL-10 and 67.0 ± 3.79 μM, n_H_ = 1.77 ± 0.16; n = 16; statistics for the dose–response experiments: *p* < 0.05 by paired t-test.

Next, we incubated oocytes microinjected with GG tissue (# 8–12; Table 1) with IL-10 (100 ng/ml) for 10 min to 4 h (Fig. 4). We observed a time-dependent increase of I_GABA_ amplitude induced by IL-10. In particular, no changes in I_GABA_ were found up to 40 min incubation, while I_GABA_ amplitude was progressively increased thereafter (+ 8.5 ± 5.4% I_GABA_ amplitude at 120 min; n = 6). The peak increase was attained after 3 h (+ 27.4% ± 3.1% I_GABA_ amplitude; n = 12; *p* < 0.001) which was maintained up to 4 h incubation (Fig. 4). On the contrary, in the subsequent experiments, we chose to incubate GG microinjected oocytes with IL-10 (100 ng/ml) for 3 h showing an average I_GABA_ increase of + 32.3 ± 2.2% (I_GABA_ = 30.35 ± 2.0 nA before IL-10 and 39.75 ± 2.8 nA after IL-10 incubation, n = 80; *p* < 0.001; # 8–12 in Table 1; Fig. 5A, B). The IL-10 effect elapsed after 1–3 h wash-out, and it was not related to a change of GABA current reversal potential (E_GABA_= − 22.9 ± 0.7 before IL-10 and − 22.4 ± 1.6 after IL-10, n = 30; # 8–12 in Table 1) nor to a change of current decay (T_0.5_ = 13.08 ± 1.7 s before vs. 14.64 ± 0.7 s after IL-10 incubation; *p* > 0.05, Wilcoxon signed rank test). IL-10 (50 ng/ml) increased I_GABA_ current to a lower extent (+ 19.1 ± 5.0%; n = 6; p < 0.05; # 8–9 in Table 1) while 200 ng/ml IL-10 increased the I_GABA_ current similarly to 100 ng/ml (+ 29.3 ± 4.2%, n = 6; *p* < 0.05; # 8–9 in Table 1). Altogether, our results show that IL-10 effect in GG was time and dose-dependent.

**Table 1:** Clinical cases used for electrophysiology and immunohistochemistry.

Pathology	Gender	Age (years)	Duration epilepsy	Seizure types	Brain area	Seizures/month	Mutation	ASMs
1. TSC	m	47y	46y	FB/TC	T	> 50	TSC2	VPA, CBZ
2. TSC	f	17y	17y	FS	F	> 50	TSC2	LEV, VPA, VGB, OXC
3. TSC	f	30y	30y	FS	F	10–20	TSC2	LMT, LEV
4. TSC	m	35y	34y	FB/TC	F	> 50	TSC2	LEV, VGB, CLB
5. TSC	m	8y	8y	FS/SE	T	20–30	TSC2	VGB, LMT, CLB
6. TSC	f	9y	9y	FS	F	> 50	TSC2	LMT, CBZ
7. TSC	m	2y	2y	FS	F	> 50	TSC2	VGB, LEV
8. GG*	m	50y	48y	FS	T	20–30	BRAF	LEV, CBZ
9. GG*	m	1y	1 m	FS	T	> 50	BRAF	LEV
10. GG*	f	23y	1y	FS	T	> 50	BRAF	LMT
11. GG	m	41y	11y	FS	T	30–40	BRAF	LMT, TPM
12. GG	m	17y	2y	FB/TC	F	20–30	BRAF	CBZ, CLB

*m* Male, *f* Female, *y* Year, *m* Month, *SE* Status epilepticus, *FS* Focal seizures, *FB/TC* Focal bilateral/tonic clonic, *F* Frontal, *T* Temporal, *CBZ* Carbamazepine, *CLB* Clobazam, LEV Levetiracetam, *LMT* Lamotrigine, *OXC* oxcarbazepine, *TPM* Topiramate, *VGB* Vigabatrin, *VPA* Valproate. Tissues from all the patients have been used to perform electrophysiology experiments with IL-10 or IL-1β. *Patients marked with asterisks have also been used to test the IL-10 after the incubation with IL-1β.

I_GABA_ amplitude was stable in the transplanted oocytes exposed only to incubation medium (see “Methods”) for 3 h showing a mean variation of -5.25% of the control amplitude (time zero, 24.8 ± 2.5 nA *versus* time 3 h, 23.5 ± 2.4 nA; n = 49; # 8–12 in Table 1). Moreover, IL-10 (100 ng/ml) did not modify I_GABA_ amplitude in oocytes transplanted with control tissues because the treatment determined a not significant average current increase of + 6.4% (I_GABA_ = 28.0 ± 4.5 nA before IL-10 and 29.8 ± 4.1 nA after IL-10, n = 17; Fig. 5A, B).

Next, we used two drugs blocking the downstream signaling activated by IL-10^27^. Both K252a, a broad spectrum protein kinases inhibitor^30^ and baricitinib, a selective JAK1 and JAK2 inhibitor^31^, pre-incubated for 30 min and co-incubated for 3 h with IL-10, prevented the cytokine effect on I_GABA_ current (Table 2 and Fig. 5C). This evidence supports that the increase of I_GABA_ amplitude induced by IL-10 in GG is mediated by the activation of IL-10 signaling axis.

**Table 2:** Effects of inhibitors of IL-10 signaling on I_GABA_ currents in GG tissues.

Treatment	n	I_GABA_ current (nA) before GABA incubation	I_GABA_ currents (nA) after GABA incubation	*p*
IL-10 (100 ng/ml)	18	31.2 ± 3.8	40.3 ± 4.1	0.01
K252a (2 μM) + IL-10	8	33.5 ± 6.0	31.9 ± 4.9	0.45
baricitinib (0.5 μM) + IL-10	8	32.2 ± 2.3	29.4 ± 2.9	0.09

Statistical significance was assessed by paired t-test; *n* Number of oocytes.

To investigate if the increase of I_GABA_ amplitude in GG was due to a change of GABA affinity, we carried out dose–response GABA experiments before and after 3 h incubation with IL-10 (100 ng/ml). We found a significant leftward shift of the GABA dose–response curve after exposure to the cytokine (GABA EC_50_ = 106.0 ± 1.5 μM, n_H_ = 1.5 ± 0.1 before IL-10 and 69.7 ± 5.0 μM, n_H_ = 1.7 ± 0.18 after IL-10; # 8–10, Table 1; n = 16; *p* < 0.05; Fig. 5D) suggesting that IL-10 induces an increase of GABA_A_R affinity.

Notably, IL-10 (100 ng/ml) incubated for 3 h with oocytes microinjected with TSC tuberal tissue did not induce a significant increase of I_GABA_ amplitude evoked by 250 μM GABA (4 s applications; 29.7 ± 4.0 nA before IL-10 and 32.4 ± 4.5 nA after IL-10 n = 24; # 1–3,5–7 in Table 1; *p* = 0.29; Fig. 5A, B). This result is compatible with the limited induction of IL-10 signaling in these specimens as compared to GG (Fig. 1).

### IL-1β effect on GABA_A_ mediated currents in GG

Since we previously showed that IL-1β inhibited GABAergic transmission in human TLE^23^, we studied whether this cytokine affects I_GABA_ amplitude in GG and TSC.

In line with our previous findings^23^, IL-1β (25 ng/mL) after 2 h incubation (peak time of effect) reduced I_GABA_ amplitude in oocytes injected with GG tissue (GABA 250 μM, 4 s applications; 52.3 ± 7.9 nA before IL-1β and 42.7 ± 7.7 nA after IL-1β; n = 17; # 8–12 in Table 1; p < 0.01). Similar results were obtained in oocytes injected with TSC cortical lesional tissues (tubers) (GABA 250 μM, 4 s applications; 52.4 ± 4.0 nA before IL-1β and 38.8 ± 3.8 nA after IL-1β; n = 22; # 1–7 in Table 1; p < 0.001). IL-1β effect was blocked by 30 min pre-incubation with 10 μM IL-1Ra, as assessed in GG (GABA 250 μM, 4 s applications; 39.6 ± 6.8 nA before IL-1β + IL-1Ra and 41.3 ± 9.0 nA after IL-1β + IL-1Ra; n = 8; # 8–11 in Table 1).

### IL-1β prevented IL-10 enhancement of GABA_A_ current

**Figure 6:**
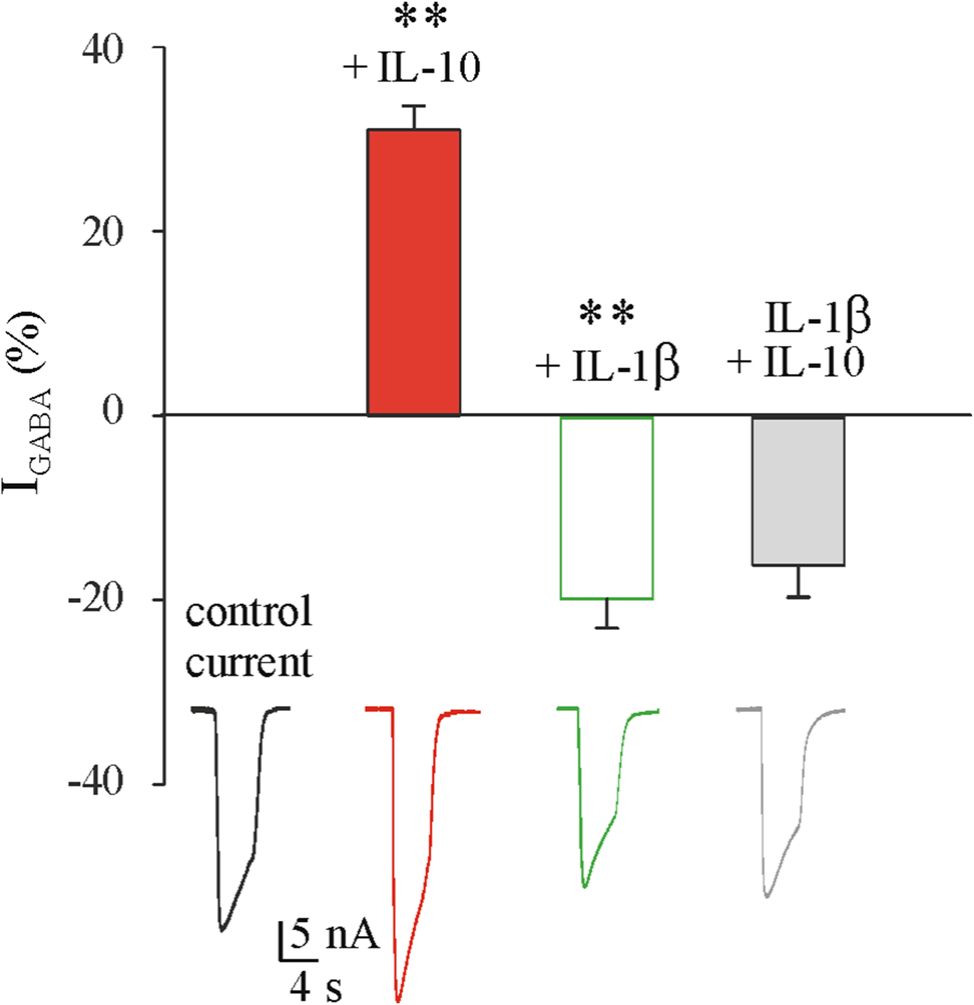
Effect of IL-1β on GABA current in the absence or presence of IL-10. Bar-graphs show the % variation in I_GABA_ amplitude induced by IL-10 (red bar and trace), IL-1β (green bar and trace) or their combination (grey bar and trace) in oocytes injected with GG tissues (Patients # 8–10; Table 1). Data are expressed as a % variation of the mean current amplitude after the incubation with each cytokine singularly or in combination. Mean current variation was + 31.0 ± 2.6% after IL-10 incubation (100 ng/ml, n = 10), − 19.6 ± 3.15% after incubation with IL-1β (25 ng/ml, n = 10) and − 15.6 ± 3.5% after co-incubation with IL-10 + IL-1β (n = 10) as described in the text. ** *p* < 0.01 by paired t-test.

We determined the net effect on GABA-evoked currents when oocytes transplanted with GG were exposed to both IL-1β and IL-10, in order to mimic the neuroinflammatory milieu of GG where both cytokines are induced with fold-increase of IL-1β exceeding that of IL-10 (Fig. 1). We pre-incubated oocytes for 30 min with IL-1β (25 ng/ml) and subsequently with a combination of IL-1β (25 ng/ml)^23^ and IL-10 (100 ng/ml) for further 3 h. IL-10 effect was suppressed by IL-1β at a concentration within the range measured in epilepsy brain tissue^32^ (GABA 250 μM, 4 s applications; 37.8 ± 5.8 nA before IL-1β + IL-10 and 31.9 ± 4.3 nA after IL-1β + IL-10; n = 10; # 8–10 in Table 1). Notably, we obtained similar results when the pre-incubation was performed with IL-10 before using the same protocol as above (55.6 ± 10.0 nA before IL-10 + IL-1β and 46.4 ± 9.4 nA after IL-10 + IL-1β; n = 9; # 8–9 in Table 1). IL-1β and IL-10 applied alone in the same set of experiments modified I_GABA_ amplitudes as expected (Fig. 6).

## Discussion

Our main objective was to study the role of the anti-inflammatory cytokine IL-10 in neurotransmission underlying GGs epileptogenicity. First, we report the novel evidence that IL-10 and related signaling molecules are up-regulated in GG, and IL-10Rα is induced in neurons and astrocytes. Similarly to GG, IL-10 receptors were induced in TSC and IL-10Rα was expressed by both dysmorphic neurons and astrocytes. However, IL-10 itself and the JAK1 downstream kinase were upregulated in GG but not in TSC, where only STAT3 was induced, suggesting that the IL-10 signaling was activated to a minor extent in TSC compared to GG. Patients with GG and TSC share common characteristics, such as a high incidence of early onset drug-resistant epilepsy and a neuroinflammatory response which is one hallmark of the neuropathology^1,5,33–36^. Notwithstanding these common features, IL-10 and related signaling have a significant impact on GABA_A_R mediated currents in GG but not in TSC, as assessed in oocytes microinjected with membranes from epileptic patients. Data support that the up-regulation of IL-10-related signaling represents a homeostatic attempt to counteract hyperexcitability in epileptogenic lesions by enhancing GABA-mediated currents. However, this up-regulation was insufficient in TSC supporting that the extent of IL-10 increase and cognate signaling activation determine the functional consequences on neurotransmission in epileptiform lesions. In support, IL-10-mediated GABA current potentiation was absent in control tissue where the cytokine and its receptor were undetectable. This evidence bears relevance for therapeutic interventions aimed at boosting IL-10R activation with stable IL-10 analogs or brain penetrant mimetic drugs^37^. A potential limitation of this study is the use of post-mortem brain tissue as control for surgical resected specimens from GG and TSC. However, comparison of the transcriptional profile has been performed demonstrating minimal variations between the two tissue types when high quality RNA was used as an input^38^. Moreover, we previously showed that surgical control tissue shows a pattern of immunoreactivity for inflammatory markers very similar to autoptic tissue, thus indicating antigen preservation in control autopsies^25,39^. In accordance, the use of post-mortem brain material is routinely used in transcriptome and immunohistochemical studies.

We used the microtransplantation approach since it allows to measure GABA currents that are otherwise difficult to record using human brain slices due to the rarity and tissue damage of surgical specimens. Indeed, to our knowledge there are no studies on ex-vivo brain slices in human GG and this is not surprising considering that GG are rare primary brain tumours with a challenging diagnosis that requires integrated diagnostic genotype–phenotype analysis, thus limiting the availability of representative tissue slices for electrophysiological recordings^1,40^. In addition, there is only one animal model of GG carrying the BRAFV600E mutation where electrophysiological studies on acute brain slices were performed, although modulation of neuronal activity by cytokines has not been evaluated yet^3,8^.

One limitation of our approach is that we microtrasplanted a mixture of glial or neuronal membranes^25^ therefore we cannot distinguish whether the IL-10 effect is mediated by glial or neuronal IL-10 receptors. However, the microtransplantation technique bypasses the biosynthetic machinery of host cell allowing the incorporation of native receptors and associated signaling that maintain their functional properties^25,41^.

Furthermore, this approach permits to use minute amounts of control tissue from individuals without neurological diseases, which is highly relevant when studying neuroinflammatory mediators.

We found that IL-10 increases GABA current amplitude in GG by activating receptor-related kinase cascade, and this effect was dose-dependent and it becomes significant after 3 h incubation. The involvement of the IL-10-related signaling is supported by (i) the blockade of the cytokine effect using drugs interfering with the downstream kinases, and (ii) the lack of IL-10 effect on GABA current in oocytes injected with exogenous cDNAs encoding human α1β2γ2 or α4β2γ2 GABA_A_Rs, thus excluding a direct interaction between IL-10 and GABA_A_Rs. In agreement, the recruitment of the same JAK/TYK signaling due to the activation of IL-10 receptor complex (IL-10Rα and IL-10Rβ) can trigger an anti-inflammatory axis that reduces neurodegenerative phenomena^18^.

Direct application of IL-10 on hippocampal naïve rat slices was reported to induce a decrease of peak amplitude and frequency of mIPSCs recorded from DG neurons^42^. This evidence is only apparently at variance with our results since we measured the IL-10 enhancing effect of GABA amplitude exclusively in pathological cortical tissues, but not in control tissues, suggesting that GABA_A_ receptor subtypes are altered by the pathology, as previously shown in epilepsy patients and animal models^43,44^.

Notably, the shift of GABA EC_50_ induced by IL-10, with no changes in reversal potential or current decay, indicates that GABA current potentiation in GG is a consequence of increased receptor affinity for GABA.

We hypothesize that IL-10 could act on tonic GABAergic inhibition which is characterized by high affinity for GABA and the activation of specific GABA_A_R subunits^29,45^. Specifically, it is likely that IL-10 could modulate the function of α4 containing GABA_A_-Rs by acting on subunit phosphorylation or receptor trafficking mechanisms^46^. Notably, in line with this hypothesis, here we blocked the IL-10 effect by using a broad spectrum kinase inhibitor. A similar role of phosphorylation was previously described for the GABA current potentiation induced by BDNF or levetiracetam in TLE patients both in oocytes and human slices^44,47,48^. In addition, we described the block of IL-10 effect with a specific inhibitor of JAK1-2 that, together with the reported lack of effect on cDNAs injected oocytes, further supports that the IL-10 signaling machinery is transplanted in the host cells.

Although our approach^25^ does not allow to determine whether the IL-10 effect is mediated by neuronal or astrocytic receptors, this aspect needs to be elucitated since enhancement of tonic extrasynaptic GABA_A_R currents may reduce seizures susceptibility^45,49^.

Previous studies showed an altered chloride homeostasis in peritumoral tissue of low-grade gliomas resulting in depolarizing GABA actions which may contribute to hyperexcitability induced by tumors^50,51^. Since we did not find any chloride alteration in GG tissues, the enhanced GABA current induced by IL-10 is likely to result in anti-ictogenic effects.

In oocytes transplanted with human TLE membranes, IL-1β reduced GABA_A_R-mediated currents through activation of its signaling pathway^26^. Our data show that IL-1β has a similar effect in GG and TSC and this effect was blocked by the specific receptor antagonist IL-1Ra confirming that it was mediated by the activation of the IL-1β receptor and associated molecular cascade as previously reported^23^. IL-1β is a key component of the neuroinflammatory milieu in epileptogenic tissue^10^, and its ability to promote neuronal NMDAR-dependent Ca^2+^ influx and decrease GABA current amplitude likely mediates its ictogenic properties. We provide novel evidence that IL-1β is induced in large excess compared to IL-1Ra in GG supporting the inefficient control of this proinflammatory signal and its pathological consequences^10^.

The contribution of cytokines to neuroinflammation in epilepsy is complex since various pro- and anti-inflammatory molecules are secreted, and they are often endowed of opposite effects on synaptic transmission and neuronal excitability^13,52,53^. In line with this scenario, our functional data show that IL-1β prevents the enhancing effect of IL-10 on GABAergic transmission while retaining its ability to decrease GABA currents, thus supporting the failure of anti-inflammatory cytokines to efficiently control neuroinflammation and the consequent hyperexcitability leading to seizures. This hypothesis is also supported by IL-10 serum levels being comparatively lower, and IFN-γ levels higher, in patients with drug-resistant *versus* drug-responsive epilepsy^10,21,54^.

Our results reinforce the link between cytokine-mediated neuroinflammation and altered neurotransmission in drug-resistant human epilepsies. In particular, our data suggest that boosting key anti-inflammatory endogenous molecules may represent a novel therapeutic strategy for controlling drug-resistant seizures as also suggested for children with febrile seizures^55^. In support, recent evidence shows that also the administration of anakinra^56,57^, the human recombinant IL-1Ra, by increasing the level of endogenous IL-1Ra provides significant therapeutic benefits in drug-resistant patients affected with febrile infection-related epilepsy syndrome.

## Conclusions

This study provides fresh evidence that the anti-inflammatory mediator IL-10 affects GABA currents in epileptogenic human tissue, thus bearing implications for novel strategies to increase inhibitory neurotransmission in drug-resistant epilepsy. Since IL-1β voided the effect of IL-10 on GABA currents, this supports that the resolution mechanisms of the pathogenic neuroinflammatory response may fail in epilepsy, thus allowing the ictogenic effects of the concurrent inflammatory molecules to prevail.

Our data provide therapeutic insights for inhibiting hyperexcitability underlying seizures by boosting endogenous anti-inflammatory homeostatic mechanisms with drugs that mimic key anti-inflammatory molecules.

## Methods

### Patients

The cases included in this study were obtained from the archives of the Departments of Neuropathology of the Amsterdam UMC (Amsterdam, the Netherlands) and the University Medical Center Utrecht (UMCU, Utrecht, the Netherlands). Cortical brain samples were obtained from patients undergoing surgery for drug-resistant epilepsy and diagnosed with GG or TSC (cortical tubers). All cases were reviewed independently by two neuropathologists, and the diagnosis of GG was confirmed according to the revised WHO classification of tumors of the central nervous system^58^. All patients with cortical tubers fulfilled the diagnostic criteria for TSC^59^. The predominant seizure types observed were focal seizures with/without impaired awareness, and all patients were resistant to maximal doses of different anti-seizure medications (ASMs) (Table 1 and Supplementary Information). All the patients included in this study had a post-surgical outcome in Engel’s class I or II. Epilepsy duration was calculated as the interval in years from the age at seizure onset to the age at tissue sampling. After resection, the tissue was immediately snap-frozen in liquid nitrogen and then part of the samples was used to perform the electrophysiology experiments. Control autopsy cases had no known history of epilepsy, a normal cortical structure for the corresponding age and no significant brain pathology. All autopsies were performed within 16–48 h after death with the acquisition of appropriate written consent for brain autopsy and subsequent use for research purposes. As pathologies in young patients are investigated, surgically resected control tissue was not available due to technical and ethical issues. The transcriptional profiles of post-mortem and surgical resected tissues have previously been compared to take into account potential post-mortem effects on RNA expression, thus showing minimal differences if the tissue is of high quality (i.e., extracted, handled and stored as in our study)^38^. Additional details can be found in Supplementary information. The brain specimens used for electrophysiological and immunohistochemical analyses are identified in the text by patient number (“#”) (Table 1). Due to the limited tissue availability of these rare human specimens, we used the frozen samples at completion for both transcriptomic analysis and electrophysiology, thus preventing additional measurements (e.g. western blot) to be done. For electrophysiological experiments, perituberal tissue was available from two TSC patients only and the amount of tissues was insufficient for recording reliable GABA currents amplitudes. Formalin-Fixed Paraffin-Embedded (FFPE) material was used for diagnostic pathology and immunohistochemistry.

Control cortical tissue was obtained from two females (age 7 yrs, intestinal ischemia; 39 yrs, respiratory failure) and one male (age 31 yrs; respiratory failure). Patients and their controls used for electrophysiological and immunohistochemical analyses (Table 1) are part of a larger cohort of GG (37 patients), TSC (21 patients) and controls (15 patients) whose tissue underwent transcriptomic analysis (Supplementary Information).

### Membrane preparation

Tissues were immediately processed upon receipt in the laboratory or stored at − 80 °C until use. Human membranes preparation, injection in *Xenopus laevis* oocytes were carried out as previously described^60,61^.

### Injection and voltage-clamp recordings

Experiments with microtransplanted oocytes were carried out 24–48 h after cytoplasmic injection^60^ (patients are reported in Table 1). GABA-evoked currents were recorded with the technique of two-electrode voltage clamp as previously reported^60^ after the oocytes were placed in a recording chamber (0.1 ml volume) and continuously perfused with oocyte Ringer solution (OR: NaCl 82.5 mM; KCl 2.5 mM; CaCl_2_ 2.5 mM; MgCl_2_ 1 mM; Hepes 5 mM, adjusted to pH 7.4 with NaOH) at room temperature (20–22 °C). These GABA currents were blocked by biculline (100 μM) as previously reported^62^, thus indicating that we recorded genuine GABA_A_ evoked responses^23^.

In one set of experiments, we used oocytes expressing human α1β2γ2 GABA_A_Rs or α4β2γ2 after intranuclear injection of cDNAs encoding human α1 or α4, β2 and γ2 GABA_A_Rs subunits^63^. cDNAs were kindly provided by Dr. K. Wafford and were used at a ratio of 1:1:1.

Unless otherwise specified, 50 μM GABA (plateau dose–response concentration) was used in the experiments with cDNA injected oocytes and 250 μM GABA (plateau dose–response concentration) with microtransplanted oocytes^63^. The stability of the evoked currents (I_GABA_) was ascertained by performing two consecutive GABA applications, separated by a 4 min washout. Only the cells that had a < 5% variation of current amplitude were used to test the effect of IL-10 and IL-1β. In some experiments we applied bicuculline (100 μM, 30 s of incubation), a competitive antagonist of GABA_A_Rs, to confirm that we recorded genuine GABA-evoked responses as previously shown^23^.

When performing dose–response relationships (before and after IL-10 incubation), we used GABA concentrations ranging from 1 μM to 1 mM, as previously reported^60^. GABA pulses were applied every 4 min to avoid receptor desensitization; to determine the half-maximal effect (EC_50_) data were fitted to Hill equations using least-square routines, as previously described^60^.

GABA current reversal potential (E_GABA_) was calculated by constructing current–voltage (I-V) relationships that were elaborated by a linear regression curve-fitting software (Sigmaplot 12, Systat software inc.). GABA current decay time (T_0.5_) was measured as the time taken for the current to decay from its peak to half-peak value after applying GABA 250 μM for 60 s^64^.

Cytokines were diluted at the final concentration (specified for each experiment) in Barth’s modified saline solution (88 mM NaCl; 1 mM KCl; 2.4 mM NaHCO_3_; 10 mM HEPES; 0.82 mM MgSO4; 0.33 mM Ca(NO_3_)_2_; 20.41 mM CaCl). IL-10 was purchased from Immunotools GmbH (Friesoythe, Germany), IL-1β was purchased from Peprotech (London, UK) and recombinant human IL-1Ra from Invitrogen (Waltham, MA, USA). Salts were purchased from Sigma-Aldrich (USA) while GABA and biculline methocloride were purchased from Tocris Bioscience (Bristol, UK) and dissolved in sterile water before dilution to the final concentration in OR.

In some experiments, we used K252a (Sigma; 2 μM), a potent non-specific inhibitor of protein kinases such as PKA, PKC, PKG and Trk receptors, and baricitinib (Selleckchem; 0.5 μM), a selective JAK1 and JAK2 inhibitor. Oocytes were incubated for 30 min with the inhibitor alone, followed by co-incubation with the cytokine for 3 h.

### Immunohistochemistry

Immunohistochemistry (patients are reported in Table 1) was carried out as previously described^65^. The primary antibody against IL-10 receptor alpha (IL-10Rα, rabbit polyclonal, Genetex, Irvine, CA, USA, 1:150) was incubated at room temperature for 1 h for single labelling. The ribosomal protein S6 was used as marker of the mTOR activity (pS6 Ser235/6, polyclonal rabbit, Cell Signaling; 1:200) to perform double-labelling of IL-10Rα.

The immunofluorescent labelling was performed as previously described^66^ by incubating NeuN (mouse monoclonal, clone MAB377; Chemicon, Temecula, CA, USA; 1:2.000), microtubule-associated protein (MAP2; mouse clone HM2; Sigma 1:100) and glial fibrillary acidic protein (GFAP; mouse monoclonal, clone GA5, Sigma-Aldrich, St. Louis, MO, USA; 1:4.000) antibody with the primary antibody against IL-10Rα. Sections were analyzed using Leica Confocal Microscope TCS SP8 X DLS (Leica, Son, the Netherlands) at 20 × magnification (bidirectional X, speed 600 Hz, pinhole 1.00 AU).

### RNA-Seq library preparation, sequencing and bioinformatics analysis

All library preparation, sequencing and bioinformatic analyses including differential expression analysis were carried out as previously described^65^ (patients are reported in Supplementary Information). Differential expression analysis compared 21 TSC patients and 15 age-matched control cortices; 37 GG patients and 15 age-matched controls cortices. The relationship between expression level of differentially expressed RNAs and subject’s age was assesssed using Spearman’s rank correlation to. A correlation coefficient of (adjusted *p* value < 0.05) > 0.7 or < − 0.7 was considered indicative of meaningful relationship between the two variables. As no significant correlation was found between gene expression levels and subject’s age, it was deemed that no correction for age needed to be applied.

### Statistics

Before data analysis, normal distribution was assessed with Shapiro–Wilk test to inform about the choice between parametric (Student's t-test) or non-parametric (Wilcoxon signed rank test, Mann–Whitney rank sum test) tests. Statistical analysis of data was performed with Sigmaplot 12 software, and differences between two data sets were considered significant when *p* < 0.05. The (n) indicates the number of oocytes used in each experiment.

### Ethics approval

Human brain tissue was obtained and used in accordance with the Declaration of Helsinki and the Amsterdam UMC Research Code provided by the Medical Ethics Committee. All the samples were used upon acquisition of appropriate written consent for research purposes. The use of *Xenopus laevis* frogs, the surgical procedures for oocytes extraction and use conformed to the Italian Ministry of Health guidelines (authorization no 427/2020-PR), and were approved by the Local Committee for Animal Health (OPBA, Department of Physiology and Pharmacology, Sapienza University). All the animal procedures followed the recommendations of the ARRIVE guidelines.

## Supplementary Information

Supplementary Information.

## Data Availability

The datasets used and/or analysed during the current study are available from the corresponding author on reasonable request.

## Abbreviations

GG: Ganglioglioma
TSC: Tuberous scleroris complex
IL-1β: Interleukin-1β
IL-10: Interleukin-10
GABA_A_: γ-Aminobutirric acid type A
BRAF: B-Raf proto-oncogene
Akt: Ak strain transforming kinase
mTOR: Mammalian target of rapamycin
NMDA: N-methyl-D-aspartate
AMPA: α-Amino-3-hydroxy-5-methyl-4-isoxazolepropionic acid
CX3CL1: Chemokine (C-X3-C motif) ligand 1
ASMs: Anti-seizure medications
IL-1Ra: Interleukin-1 receptor antagonist
pS6: S6 ribosomal protein
TYK2: Tyrosine kinase 2
PKA: Protein kinase A
PKC: Protein kinase C
PKG: Protein kinase G
CNS: Central nervous system
I_GABA_: GABA current
E_GABA_: GABA current reversal potential
I-V: Current–voltage relationship
JAK1: Janus activated kinase 1
JAK2: Janus activated kinase 2
IL-10Rα: Interleukin-10 receptor α
IL-10Rβ: Interleukin-10 receptor β
NeuN: Neuronal nuclei antigen
MAP2: Microtubule-associated protein 2
GFAP: Glial fibrillary acidic protein
STAT3: Signal transducer and activator of transcription 3
mIPSCs: Miniature inhibitory post-synaptic currents
DG: Dentate gyrus neurons
EC_50_: Half maximal effective concentration
IFN-γ: Interferon γ
