# Supplementary material for: GABAA receptor function is enhanced by Interleukin-10 in human epileptogenic gangliogliomas and its effect is counteracted by Interleukin-1β
Source: Sci Rep. 2022 Oct 26;12:17956. doi: 10.1038/s41598-022-22806-9 (PMC9605959; doi:10.1038/s41598-022-22806-9)
Supplement: Supplementary file 1 — Supplementary Information. [file 41598_2022_22806_MOESM1_ESM.docx]

**Supplementary Information**

| **ID** | **Pathol.** | **Gender** | **Age-years** | **Epilepsy**  **duration-years** | **Seizure Type** | **Subclassification Seizures** | **Brain Area** | **ASMs** | **Mut.** |
| --- | --- | --- | --- | --- | --- | --- | --- | --- | --- |
| 1* | TSC | f | 8 | 8 | FS/SE | Impaired Awareness | T | VGB,LMT,CLB | TSC2 |
| 2* | TSC | m | 2 | 2 | FS | Impaired Awareness | F | LEV, VGB | TSC2 |
| 3 | TSC | f | 21 | 1 | FS | Impaired Awareness | T | VPA | TSC1 |
| 4* | TSC | m | 35 | 34 | FB/TC | Impaired Awareness | F | LEV, VGB,CLB | TSC2 |
| 5 | TSC | m | 4 | 4 | FS | Impaired Awareness | T | CLB, VPA, VGB | TSC1 |
| 6 | TSC | f | 2 | 2 | FS | Impaired Awareness | T | CNP, LEV, OXC, ZNS | TSC1 |
| 7 | TSC | m | 2 | 2 | FS | Impaired Awareness | F | ZNS | TSC2 |
| 8 | TSC | m | 2 | 2 | FS | Impaired Awareness | F | CLB, LEV, PB, VGB | TSC2 |
| 9 | TSC | m | 1 | 1 | FS | Impaired Awareness | F | CLB, VGB, ZNS | TSC2 |
| 10 | TSC | m | 0 | 0 | FS | Impaired Awareness | F | CLB, LEV, VGB | TSC2 |
| 11* | TSC | m | 47 | 46 | FB/TC | Impaired Awareness | T | VPA, CBZ | TSC2 |
| 12 | TSC | m | 0 | 0 | FS | Aware | F | CBZ, LEV, VGB | TSC2 |
| 13 | TSC | m | 3 | 1 | FS | Impaired Awareness | F | LEV | TSC1 |
| 14* | TSC | f | 9 | 9 | FS | Impaired Awareness | F | VGB,LEV | TSC2 |
| 15 | TSC | f | 9 | 1 | FS | Impaired Awareness | F | CLB, OXC | TSC2 |
| 16 | TSC | v | 0 | 0 | FS | Impaired Awareness | F | CLB, LEV, VGB | TSC2 |
| 17* | TSC | f | 30 | 4 | FS | Aware | F | LMT, LEV | TSC2 |
| 18 | TSC | f | 0 | 0 | FS | Impaired Awareness | T | LEV, OXC, VGB | TSC2 |
| 19* | TSC | f | 17 | 17 | FS | Impaired Awareness | F | LEV, OXC, VPA, VGB | TSC2 |
| 20 | TSC | f | 11 | 6 | FS | Impaired Awareness | T | CLB, TPM, VGB | TSC2 |
| 21 | TSC | m | 4 | 4 | FS | Impaired Awareness | F | CLB, VGB, ZNS | TSC2 |
| 22 | GG | m | 44 | 24 | FB/TC | Impaired Awareness | T | OXC | NA |
| 23 | GG | f | 6 | 5 | FS | Impaired Awareness | T | OXC | NA |
| 24 | GG | m | 34 | 12 | FB/TC | Impaired Awareness | T | PNT | NA |
| 25 | GG | m | 33 | 15 | FS | Impaired Awareness | T | LMT, OXC | BRAF |
| 26 | GG | m | 10 | 0 | FS | Impaired Awareness | O | PNT | NA |
| 27 | GG | m | 28 | 0 | FS | Aware | P | OXC, PNT | NA |
| 28 | GG | m | 9 | 1 | FS | Impaired Awareness | T | CBZ | BRAF |
| 29 | GG | f | 28 | 5 | FB/TC | Impaired Awareness | T | LEV, OXC | BRAF |
| 30 | GG | f | 24 | 0 | FS | Impaired Awareness | T |  | NA |
| 31 | GG | m | 42 | 3 | FB/TC | Impaired Awareness | T | CLB, LEV, OXC | NA |
| 32 | GG | m | 16 | 4 | FB/TC | Impaired Awareness | P | LMT, LEV | NA |
| 33 | GG | m | 41 | 11 | FS | Impaired Awareness | T | LMT, TPM | NA |
| 34 | GG | m | 26 | 6 | FB/TC | Impaired Awareness | F | LMT, OXC | NA |
| 35 | GG | f | 3 | 2 | FS | Impaired Awareness | T | AAZ, CLB, ETX, LMT, OXC | NA |
| 36 | GG | m | 21 | 14 | FS | Impaired Awareness | P | LMT, OXC | NA |
| 37 | GG | f | 14 | 12 | FS | Impaired Awareness | F | CLB, OXC, TPM | NA |
| 38 | GG | f | 6 | 3 | FS | Impaired Awareness | T | OXC | BRAF |
| 39 | GG | m | 1 | 1 | FS | Impaired Awareness | T | CLB, LEV | BRAF |
| 40 | GG | m | 3 | 1 | FS | Impaired Awareness | T | CBZ, CLB, LEV | NA |
| 41* | GG | f | 23 | 1 | FS | Impaired Awareness | T | LMT | BRAF |
| 42* | GG | m | 50 | 48 | FS | Aware | T | CBZ, LEV | BRAF |
| 43 | GG | m | 13 | 11 | FB/TC | Aware | T | CBZ, VPA, VGB | BRAF |
| 44 | GG | m | 5 | 0 | FS | Impaired Awareness | T | CBZ, LEV | BRAF |
| 45 | GG | f | 8 | 0 | FS | Impaired Awareness | T | CBZ, LEV | NA |
| 46 | GG | m | 14 | 4 | FS | Impaired Awareness | F | CBZ | BRAF |
| 47 | GG | m | 4 | 3 | FS | Impaired Awareness | T | CBZ | NA |
| 48 | GG | m | 3 | 0 | FS | Impaired Awareness | T | LEV | NA |
| 49 | GG | f | 11 | 1 | FS | Impaired Awareness | T | CBZ, PB | BRAF |
| 50 | GG | f | 18 | 12 | FS | Impaired Awareness | T | CBZ, GBP, OXC | BRAF |
| 51 | GG | f | 13 | 1 | FS | Impaired Awareness | T | CBZ | BRAF |
| 52 | GG | m | 12 | 1 | FS | Impaired Awareness | T | CBZ, PB | BRAF |
| 53 | GG | f | 13 | 0 | FS | Aware | F | CBZ | NA |
| 54 | GG | m | 4 | 2 | FS | Impaired Awareness | T | CBZ, VPA | BRAF |
| 55 | GG | m | 1 | 1 | FS | Impaired Awareness | T | LEV | NA |
| 56 | GG | f | 13 | 0 | FS | Impaired Awareness | F | CBZ, VPA | NA |
| 57 | GG | m | 11 | 4 | FS | Aware | T | CBZ, ZNS | NA |
| 58 | GG | f | 2 | 2 | FS | Impaired Awareness | T | CBZ | BRAF |
| 59* | GG | m | 1 | 1month | FS | NA | T | LEV | BRAF |
| 60* | GG | m | 41 | 11 | FS | NA | T | LMT, TPM | BRAF |
| 61* | GG | m | 17 | 2 | FB/TC | NA | F | CBZ, CLB | BRAF |
| 62 | Control | f | 0 | NA | NA | NA | C | NA | NA |
| 63 | Control | m | 10 | NA | NA | NA | C | NA | NA |
| 64 | Control | f | 0 | NA | NA | NA | C | NA | NA |
| 65 | Control | f | 2 | NA | NA | NA | C | NA | NA |
| 66 | Control | m | 15 | NA | NA | NA | C | NA | NA |
| 67 | Control | m | 13 | NA | NA | NA | C | NA | NA |
| 68 | Control | f | 0 | NA | NA | NA | C | NA | NA |
| 69 | Control | m | 10 | NA | NA | NA | C | NA | NA |
| 70 | Control | f | 17 | NA | NA | NA | C | NA | NA |
| 71 | Control | f | 17 | NA | NA | NA | C | NA | NA |
| 72 | Control | f | 61 | NA | NA | NA | C | NA | NA |
| 73 | Control | f | 44 | NA | NA | NA | C | NA | NA |
| 74 | Control | m | 60 | NA | NA | NA | C | NA | NA |
| 75 | Control | f | 39 | NA | NA | NA | C | NA | NA |
| 76 | Control | m | 49 | NA | NA | NA | C | NA | NA |

m = male, f = female, ASMs = anti-seizures medications, Pathol.= pathology, Mut.= mutations, SE = status epilepticus, FS = focal seizures, FB/TC = focal bilateral/tonic clonic, C=Cortex, F=Frontal, O=Occipital, P=Parietal, T=Temporal, AAZ = Acetazolamide, CBZ = Carbamazepine, CLB = Clobazam, CNP = Clonazepam, ETX = Ethosuximide, GBP = Gabapentin, LEV = Levetiracetam, LMT = Lamotrigine, OXC = Oxcarbazepine, PB = Phenobarbital, PNT = Phenytoin, TPM =Topiramate, VGB = Vigabatrin, VPA = Valproate, ZNS = Zonisamide. The samples marked with an asterisk have been used to perform electrophysiology experiments.

**Supplementary Figure**

**Fig. 1.** Semiquantitative analysis of IL-10Ra immunoreactivity in neurons and astrocytes in GG (blue, n = 9) and in neurons, astrocytes and giant cells in TSC (red, n = 6) compared to neuronal and astroglial expression in control cortex (n = 6). Tukey’s multiple comparisons test was performed to determine the significance: * p ≤ 0.05; ** p ≤ 0.01; *** p ≤ 0.001; **** p ≤ 0.0001. Immunoreactivity score (IRS); Tuberous sclerosis complex (TSC); ganglioglioma (GG).

**Supplementary Materials and Methods**

*Evaluation of immunoreactivity*

Semiquantitative evaluation of immunoreactivity was performed for IL-10Rα immunostaining using an Olympus microscope and examining each section with high-power non overlapping fields (of 0.0655 mm × 0.0655 mm width; each corresponding to 4.290 μm^2^; using a square grid inserted into the eyepiece). The staining intensity of the immunoreactive signal was evaluated in neurons, astrocytes and giant cells using a scale of 1–4 (1: no; 2: weak; 3: moderate; 4: strong signal). This score represents the predominant staining intensity found as averaged from the selected fields. Furthermore, the relative number of positive cells (0: no; 1: single to 10%; 2:11–50%; 3: >50%) was also evaluated in these areas. Then the IRS was calculated by multiplying the intensity score by the relative number score. Tukey’s multiple comparisons test was performed to determine the significance: * p ≤ 0.05; ** p ≤ 0.01; *** p ≤ 0.001; **** p ≤ 0.0001.
